# Association of visceral fat and plasmacytoid dendritic cell-derived interferon alpha with SARS-CoV-2 infection

**DOI:** 10.1371/journal.pone.0344870

**Published:** 2026-04-10

**Authors:** Naoki Ozato, Shota Hori, Nobuyuki Miyai, Hideto Takase, Ryohei Tsuji, Noriko Osaki, Daisuke Fujiwara, Mikio Arita

**Affiliations:** 1 Human Health Care Products Research Laboratories, Kao Corporation, Tokyo, Japan; 2 Institute of Health Sciences, Kirin Holdings, Co., Ltd., Kanagawa, Japan; 3 Graduate School of Health and Nursing Science, Wakayama Medical University, Japan; 4 Department of Cardiology, Sumiya Rehabilitation Hospital, Wakayama, Japan; University of Illinois, UNITED STATES OF AMERICA

## Abstract

Type I interferon (IFN-I: IFNα/β) production by plasmacytoid dendritic cells (pDCs) is critical for controlling viral infections. Visceral fat obesity is associated with severe outcomes in viral infections, but its effect on pDC-mediated IFN-I production in the context of SARS-CoV-2 infection remains unclear. This cross-sectional study evaluated cytokine production by blood pDCs in response to TLR7/8 stimulation and analyzed its association with visceral fat area (VFA). IFN-I production was exclusively observed in pDCs following TLR7/8 stimulation. The proportion of IFNα-positive pDCs (pDC-IFNα) was significantly lower in individuals with high VFA (above the median) and negatively correlated with VFA (r = −0.27, P < 0.001). Logistic regression revealed that high VFA or low pDC-IFNα levels (below the median) were independently associated with a history of SARS-CoV-2 infection. Moreover, individuals with both high VFA and low pDC-IFNα levels had significantly higher odds of infection compared to those with low VFA and high pDC-IFNα levels. These findings suggest a potential relationship between VFA and impaired pDC function, which may influence susceptibility to SARS-CoV-2 infection. Managing VFA and enhancing pDC responses might represent strategies to reduce infection risk in individuals with visceral fat obesity.

## Introduction

The recent coronavirus disease (COVID-19), caused by severe acute respiratory syndrome coronavirus 2 (SARS-CoV-2) [1], and influenza [2,3] pandemics have resulted in a considerably increased global interest in preventive measures against infectious diseases. Given the unpredictable nature of these epidemics and pandemics, it is crucial to devise strategies to reduce their impact [3].

Obesity, commonly assessed using body mass index (BMI), is a well-established risk factor for increased severity of SARS-CoV-2 infection [4] and influenza [5,6]. However, epidemiological studies have shown that the magnitude of obesity-associated risk differs substantially across populations; for example, obesity is associated with a more than five-fold increase in the risk of severe SARS-CoV-2 infection in Asian populations, compared with an approximately two-fold increase in Western populations, relative to non-obese individuals within each population [7]. These population-dependent differences suggest that BMI alone may not adequately capture obesity-related risk for severe viral infections. BMI does not distinguish between visceral and subcutaneous fat accumulation, nor does it reflect fat distribution, which varies across ethnic groups and has been shown to influence susceptibility to severe SARS-CoV-2 infection [8,9]. In addition to BMI, various indicators of obesity, including visceral and subcutaneous fat obesity, have been proposed. Asians have a high incidence of visceral fat obesity; a meta-analysis study reported that visceral fat obesity, but not subcutaneous fat obesity, was significantly correlated with the severity of SARS-CoV-2 infection [8]. Furthermore, a recent study confirmed that among obesity-related indicators, including the BMI and subcutaneous fat content, visceral fat is the best prognostic indicator of SARS-CoV-2 infection [9]. In addition, an epidemiological study reported that visceral obesity, and not the BMI, is associated with the occurrence of influenza infection in Asians [10]. These results suggest that visceral fat may be a cause of this phenomenon, and appropriate management of the visceral fat area (VFA) might play an important role in the prevention of unpredictable infectious diseases.

Type I interferons (IFN-I; IFNα/β) are essential mediators of antiviral immunity, acting through the interferon-alpha/beta receptor (IFNAR) to impact both hematopoietic and non-hematopoietic cells [11]. In hematopoietic cells, IFN-I enhances the activation of key immune components, such as dendritic cells, macrophages, and natural killer cells, thereby strengthening their ability to detect and eliminate infected cells. IFN-I also promotes the differentiation of CD8 + cytotoxic T cells and supports the formation of memory T cells [12,13], thereby shaping the adaptive immune response. In parallel, IFN-I induces the expression of interferon-stimulated genes (ISGs) in non-hematopoietic cells, establishing an antiviral state that inhibits viral replication, transcription, and assembly [14,15]. By coordinating the activation of both cell types, type I IFNs connect innate and adaptive immunity, leading to a robust and multifaceted defense against viral pathogens.

Although nearly all hematopoietic and non-hematopoietic cells can produce IFN-I in response to viral infections, these cytokines, particularly IFN-α, are primarily produced by plasmacytoid dendritic cells (pDCs) in both humans and mice [16–19]. pDCs possess a unique ability to rapidly produce large quantities of IFNα upon recognizing viral components through highly expressed pattern recognition receptors, for example, Toll-like receptor 7 (TLR7) for single-stranded RNA (ssRNA) [20,21] and TLR9 for DNA [22,23]. This early and robust production of IFNα by pDCs is critical for initiating the antiviral state in host cells, enhancing the recruitment and activation of other immune cells and coordinating both innate and adaptive immune responses to effectively control viral replication.

Visceral fat accumulation is linked to chronic low-grade inflammation and an altered immune response, which may contribute to a dysregulated pDC-IFNα response during SARS-CoV-2 infection. Decreased IFNα production is observed in peripheral blood mononuclear cells (PBMCs) of individuals with obesity [24], and recent studies have reported reduced serum IFNα levels in obese patients infected with SARS-CoV-2 [25]. Furthermore, a human clinical study reported a correlation between impaired pDC-derived -IFNα production and the severity of SARS-CoV-2 infection [26]. Despite these findings, the relationship between pDC-derived IFNα, visceral fat, and viral infections remains poorly understood. Thus, investigating the role of pDCs and their capacity to produce IFNα in obese individuals could provide critical insights into the mechanisms underlying the increased susceptibility to severe COVID-19 in individuals with obesity.

In this cross-sectional study of 223 individuals, we aimed to investigate the association between visceral fat accumulation and pDC-derived IFNα production, and to examine whether these factors are associated with susceptibility to SARS-CoV-2 infection. Given the established roles of visceral fat in immune dysregulation and of pDC-derived IFNα in antiviral defense, we hypothesized that increased visceral fat and impaired pDC-IFNα production may synergistically contribute to increased susceptibility to SARS-CoV-2 infection.

## Materials and methods

### Study participants

This cross-sectional study examined participants of the Wakayama Health Promotion Study (the Wakayama Study). The Wakayama Study is a community-based prospective cohort study that began in 2011. The participants were middle-aged and elderly residents of rural districts in Wakayama, western Japan [27,28]. VFA and IFN-I levels in pDCs were first introduced as health checkup parameters in 2022. The present analyses were performed using data obtained from the 2022 health checkup as a population-based cross-sectional study. Participants presenting with symptoms of acute infection on the day of blood collection were excluded from the study. Due to limitations in measurements of IFNα levels in pDCs, analyses were restricted to individuals aged 50–55 years. PBMCs from these donors were stimulated with a TLR7/8 ligand, as described in the “Cell Stimulation and Flow Cytometry” section. A total of 223 individuals were included in the analysis. Each donor sample was stimulated once due to limited PBMC availability, and no technical replicates were performed. Thus, the total number of analyzed samples corresponds to 223 independent biological replicates (donors). Written informed consent for the use of examination data was obtained from all participants. This study was approved by the ethics committee of Wakayama Medical University (approval no. 92) and conducted in accordance with the principles of the Declaration of Helsinki.

### SARS-CoV-2 infection or influenza

SARS-CoV-2 infection or influenza was assessed using a self-administered questionnaire according to a previous study [29]. Participants were asked if they had SARS-CoV-2 infection or influenza in the past year (yes/no). They were expected to answer the questionnaire based on the doctor’s diagnosis using a commercial rapid diagnostic test kit, which has a high sensitivity (97.1%) and specificity (89.2%) and is commonly used in clinical settings such as outpatient clinics in Japan [30].

### VFA measurement

The VFA was measured using a bioimpedance-type visceral fat meter (EW-FA90; Panasonic Corporation, Osaka, Japan), which is a certified medical device in Japan (No. 22500BZX00522000) for the non-invasive measurement of VFA [31]. Measurements obtained using this device have been reported to be strongly correlated with those obtained using computed tomography, which is the gold standard for VFA measurement [32].

### Isolation of human peripheral blood mononuclear cells

Blood was drawn directly into BD Vacutainer® CPT™ Mononuclear Cell Preparation Tubes (CPT) (Becton Dickinson, Franklin Lakes, USA), and PBMCs were isolated according to the manufacturer’s instructions. The collected PBMCs were mixed gently with 1 mL CELLBANKER 2 (Zenogen Pharma, Fukushima, Japan), transferred to cryotubes, and gradually frozen in frosty containers (BICELL, Nihon Freezer, Tokyo, Japan) at −80 ℃. After 24 h, the cryotubes were transferred to LN2 storage until analysis.

### Cell stimulation and flow cytometry

On the day of analysis, frozen PBMCs were thawed and resuspended in RPMI 1640 medium (Sigma-Aldrich, St. Louis, United States) with 10% fetal bovine serum, 1% non-essential amino acids (Gibco, Carlsbad, United States), and 1% penicillin/streptomycin (Gibco). After removing the supernatant, cells were filtered through a 45-μm filter in an FACS tube, centrifuged, and adjusted to 1.5 × 10^6^ cells/mL in 12-well plates. PBMCs were incubated with 10 μM R848 (InvivoGen, Toulouse, France) for 4 h at 37 °C/5% CO_2_ to evaluate TLR7/8 signaling relevant to RNA viruses, including SARS-CoV-2 and influenza [33,34]. R848 preferentially activates TLR7 and induces robust IFN-I production [35], enabling functional assessment of pDC antiviral responses [36]. Brefeldin A (BioLegend, San Diego, United States) was added for the final 2 h to retain intracellular cytokines. Cells were collected, washed with PBS, stained with Zombie Red Fixable Viability Kit (BioLegend) in PBS and washed in FACS buffer (PBS + 0.5% BSA). After Fc block (BD Pharmingen, San Diego, United States), cells were stained for extracellular markers with Lineage Cocktail FITC (CD3, CD14, CD19, CD20, CD56; BioLegend), CD11c PE/Cyanine7 (BioLegend), HLA-DR APC-H7 (BD), CD123 PerCP/Cyanine5.5 (BioLegend), and CD303 Brilliant Violet 421™ (BioLegend) for 30 min at room temperature and then washed twice. After fixation/permeabilization (BD), cells were stained for intracellular cytokines with TNF-α Brilliant Violet 510™ (BioLegend), IFN-α[2b] PE (BD), and IL-6 Alexa Fluor™ 700 (eBioscience, California, United States) in Perm buffer for 20 min, followed by washing.

Cells were resuspended in 300 μL FACS buffer and analyzed using a Navios EX flow cytometer (Beckman Coulter, California, United States) and Kaluza software. The gating strategy is shown in S1 Fig. pDCs were defined as live lineage⁻ HLA-DR ⁺ CD11c ⁻ CD123 ⁺ cells, and mDCs were defined as live lineage⁻ HLA-DR ⁺ CD11c ⁺ CD123 ⁻ cells. Non-pDCs were defined as live CD123 ⁻ CD303 ⁻ cells after exclusion of dead cells. Intracellular cytokine production (IFN-α, TNF-α, and IL-6) was then quantified, with fluorescence minus one, isotype controls, and unstimulated full-stain controls to confirm appropriate gating. Non-stimulated samples showed approximately 0.1% cytokine-positive cells (S1 Fig).

### Measurement of other parameters

All measurements were performed in the morning after overnight fasting. The participants were instructed to refrain from vigorous physical activity, smoking, and drinking caffeinated beverages before examination. The following clinical characteristics were measured: body weight, height; BMI, waist circumference, diastolic blood pressure (DBP), systolic blood pressure (SBP), glycated hemoglobin, fasting serum glucose, total serum cholesterol concentration, triglycerides; LDL cholesterol, and HDL cholesterol. All laboratory tests were outsourced to LSI Medience Co. (Tokyo, Japan). Blood samples were collected in the morning from the peripheral veins of participants. Smoking habits, exercise habits, and education were determined using a questionnaire. Current smoking was defined as habitual smoking at the time of the survey, defined as having smoked ≥100 cigarettes in total or for ≥6 months and having smoked within the past month. Regular exercise was defined as engaging in light-to-moderate physical activity causing mild sweating for ≥30 minutes per session at least twice per week for ≥1 year. Education level was categorized as college or higher versus lower education. Hypertension was defined as SBP ≥ 130 mmHg, DBP ≥ 85 mmHg, or current use of antihypertensive medication. Hyperglycemia was defined as fasting blood glucose ≥110 mg/dL or current use of antidiabetic medication. Dyslipidemia was defined as TG ≥ 150 mg/dL, HDL-C < 40 mg/dL, or current use of antihyperlipidemic medication.

### Statistical analysis

Participant characteristics are presented as mean ± standard deviation. Groups (high-VFA vs low-VFA, and high-pDC-IFNα vs low-pDC-IFNα) were compared using the Wilcoxon rank-sum test, one-way ANOVA, and Fisher’s exact test. The association between VFA and pDC-IFN production was assessed using Spearman’s correlation coefficient. The association between VFA and pDC-IFNα production was evaluated using a linear regression model, considering pDC-IFNα production as an objective variable and VFA and covariates, including age, sex, and lifestyle habits, as explanatory variables. To evaluate whether the association between VFA and pDC-derived IFNα production differed by sex, a sex-by-VFA interaction term was included in a multivariable linear regression model, with pDC-derived IFNα production as the dependent variable and VFA, sex, their interaction term, and the same covariates used in the primary linear regression analysis of VFA and pDC-derived IFNα production as independent variables. The association of the incidence of SARS-CoV-2 infection or influenza with VFA or pDC-IFNα status was assessed using a logistic regression model, with age, sex, muscle mass, education, smoking habit, BMI, exercise habits, alcohol consumption, corresponding vaccine doses (number of SARS-CoV-2 or influenza vaccinations), and prevalent confounding diseases (hypertension, hyperlipidemia, and hyperglycemia) included as covariates. We calculated ORs and 95% confidence intervals based on the model.

All statistical tests were two-tailed. P-values < 0.05 were considered statistically significant. We used the R statistical software and environment (version 4.0.5) for statistical analyses.

## Results

### Characteristics of individuals

The participants were divided into two groups based on the median VFA values (low VFA vs high VFA) or pDC-IFNα levels (low pDC-IFNα vs high pDC-IFNα). The characteristics of the participants (N = 223, 60% female), stratified by VFA and pDC-IFNα, are summarized in Tables 1 and 2 and S1 and S2 Tables. The overall proportion of overweight individuals (BMI ≥ 25 kg per m^2^) was 33.3% for men and 14.3% for women. The obesity rate (BMI ≥ 30 kg per m^2^) was 2.2% for men and 6.8% for women. These rates are comparable to those reported in the 2010 Japanese national survey (overweight and obesity rates in individuals 30–69 years of age: 33.5% for men and 20.5% for women) [Ministry of Health, Labour and Welfare 2011 Japan National Health and Nutrition Survey 2010. (Japan: MHLW)]. The mean VFA was 84.2 ± 46.3 cm^2^, which is lower than the value defined as visceral obesity (≥100 cm^2^) by the Japan Society for the Study of Obesity [37]. Additional metabolic and lifestyle characteristics are summarized in S1 and S2 Tables. A simple comparative analysis with no confounder adjustment revealed that the high-VFA group had significantly higher cardiometabolic risk markers, including systolic and diastolic blood pressure, serum glucose, hemoglobin A1c (HbA1C), and C-reactive protein (CRP) levels, than the low-VFA group (all P < 0.001). Furthermore, the high-VFA group consumed significantly more alcohol and smoked more than the low-VFA group (P = 0.041 and 0.005 for smoking and drinking, respectively). In contrast, when stratified by pDC-IFNα levels, the high-pDC-IFN-α group had significantly higher systolic blood pressure and HbA1C levels than the low-pDC-IFNα group (P = 0.008 and 0.017, respectively), whereas other metabolic and lifestyle parameters did not differ substantially between the groups.

**Table 1:** Characteristics of the participants divided into two groups based on the median values of VFA.

	Low-VFA	High-VFA	P value^a, b^
n	114	109	
Age (mean (SD))	53.0 (1.4)	53.0 (1.5)	0.799
Sex (n, %)			<0.001^***^
Male	15 (13%)	75 (69%)	
Female	99 (87%)	34 (31%)	
BMI (mean (SD))	20.36 (2.06)	25.28 (3.10)	<0.001^***^
Muscle mass (mean (SD))	16.52 (3.82)	23.90 (4.79)	<0.001^***^
COVID-19 vaccination (Yes) (n, %)	103 (90.4%)	99 (90.8%)	0.732
COVID-19 vaccination doses (mean (SD))	3.07 (1.12)	3.13 (1.21)	0.372

* P < 0.05; ** P < 0.01; *** P < 0.001. Data represent mean (standard deviation). P-values were evaluated between the low-VFA and high-VFA groups.

^a^ Wilcoxon rank-sum test was performed. ^b^ Fisher’s exact test was performed.

VFA, visceral fat area.

**Table 2:** Characteristics of the participants divided into two groups based on the median values of pDC-IFNa.

	Low-pDC_IFNa	High-pDC_IFNa	P value ^a, b^
n	112	111	
Age (mean (SD))	53.0 (1.461)	53.1 (1.4)	0.613
Sex (n, %)			0.086
Male	52 (46%)	38 (34%)	
Female	60 (54%)	73 (66%)	
BMI (mean (SD))	23.20 (3.50)	22.32 (3.65)	0.079
Muscle mass (mean (SD))	21.22 (5.77)	19.07 (5.41)	0.004^**^
COVID-19 vaccination (Yes) (n, %)	105 (93.8%)	97 (87.4%)	0.147
COVID-19 vaccination doses (mean (SD))	3.10 (1.11)	3.10 (1.22)	0.643

* P < 0.05; ** P < 0.01; *** P < 0.001. Data represent mean (standard deviation). P-values were evaluated between the low-pDC and high-pDC-IFNα groups.

^a^ Wilcoxon rank-sum test was performed. ^b^ Fisher’s exact test was performed.

pDC-IFNα, plasmacytoid dendritic cell-derived interferon alpha.

### Relationship between visceral adipose area and pDC-IFNα production in response to R848

**Fig 1:**
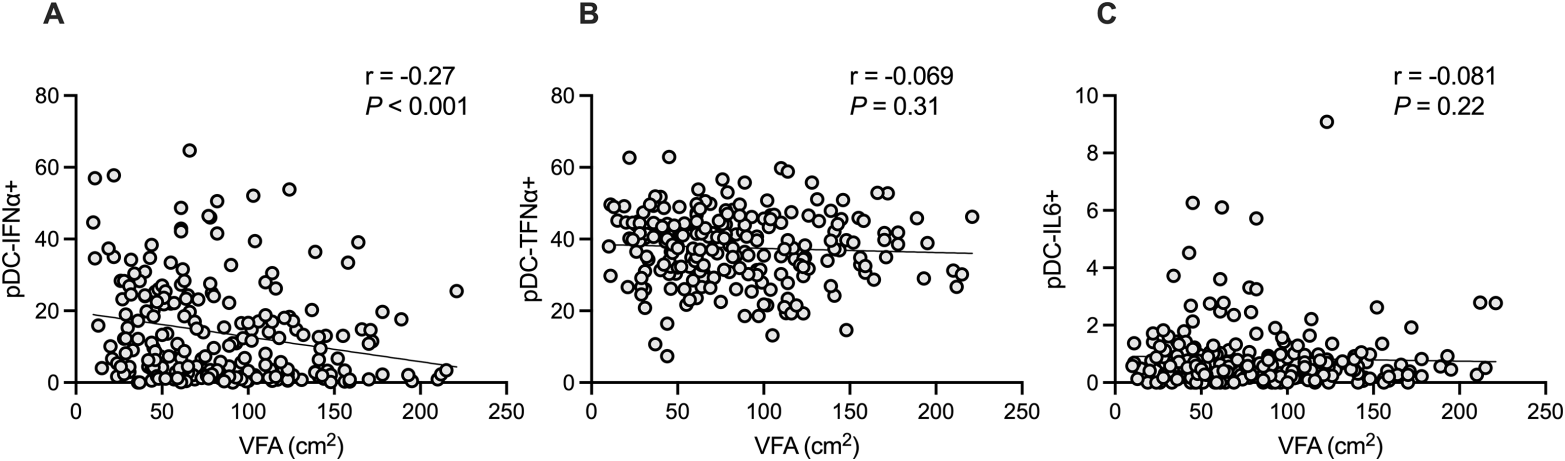
Association between the VFA and pDC-IFNα (n = 223). Scatter plot with linear regression lines showing the relationship between VFA and pDC-IFNα (A), pDC-TNFa (B), and pDC-IL6 (C). Correlation between the two variables was determined using Spearman’s correlation coefficient. VFA, visceral fat area; pDC-IFNα, plasmacytoid dendritic cell-derived interferon alpha; pDC-TNFα, plasmacytoid dendritic cell-derived tumor necrosis factor alpha; pDC-IL6, plasmacytoid dendritic cell-derived interleukin 6.

Visceral fat obesity is associated with an increased incidence of various infectious diseases, including SARS-CoV-2 infection and influenza. Given the importance of pDC-derived IFN-I in controlling viral infections, we hypothesized that individuals with high VFA exhibit lower IFN-α production from pDCs in response to TLR7 sensing than those with low VFA. PBMCs isolated from participants were cultured in the presence of TLR7/8 agonist R848 for 4 h, with the last 2 h in the presence of brefeldin A to retain the intracellular cytokines. Intracellular cytokine production was then analyzed using multi-color flow cytometry. S1 Fig shows the gating strategy. The percentage of pDC-IFN + α was negatively correlated with the VFA (r = −0.27, P < 0.001, Fig 1A), whereas that of pDC-derived tumor necrosis factor alpha (pDC-TNFα+) and pDC-derived interleukin 6 (pDC-IL6+) was not correlated with VFA (Fig 1B-1C). When stratified by sex, a significant negative correlation between VFA and pDC-derived IFNα production was observed in females (r = −0.27, P = 0.001; S3A in S3 Fig), whereas no significant correlation was detected in males (r = −0.06, P = 0.57; S3B in S3 Fig). However, formal interaction analysis did not identify a significant sex-by-VFA interaction.

**Fig 2:**
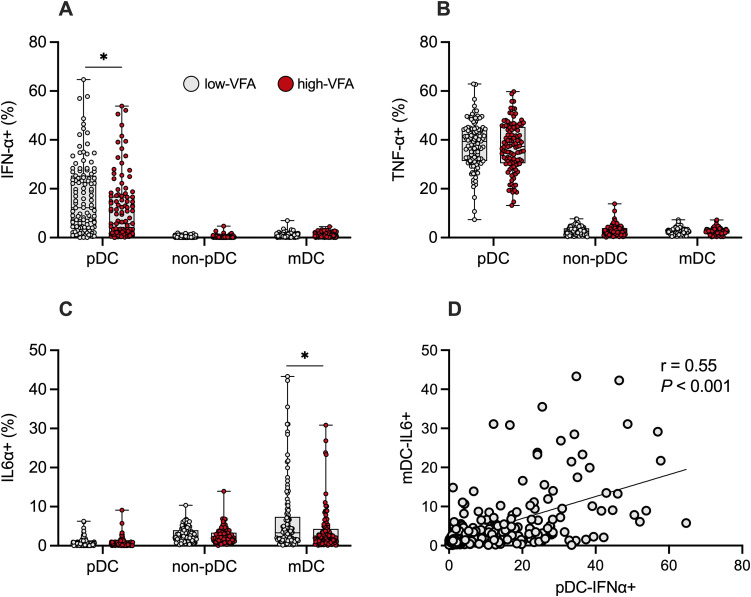
Cytokine production of pDC, non-pDC, and mDC in the low- and high-VFA groups. Percentages of IFNα+ (A), TNFα+ (B), and IL6+ (C) pDCs, non-pDCs, and mDCs in the low- and high-VFA groups. Data are presented as box-and-whisker plots (center line, median; box limits, upper and lower quartiles; whiskers, min to max; points, individual data). Differences between groups were evaluated using one-way ANOVA. ** P < 0.01. (D) Scatter plot with linear regression lines showing the relationship between the percentage of pDC-IFNα+ and mDC-IL6 + . Correlation between two variables was determined using Spearman’s correlation coefficient. VFA, visceral fat area; pDC-IFNα, plasmacytoid dendritic cell-derived interferon alpha; mDC, myeloid dendritic cell; pDC, plasmacytoid dendritic cell.

The percentage of pDC-IFNα+ in response to R848 treatment was significantly lower in the high VFA group than in the low VFA group (Fig 2A). There was no significant difference in IFNα+ between the high- and low-VFA groups in mDCs and non-pDCs (Fig 2A). Furthermore, there was no difference in the percentage of TNFα+ in pDC, non-pDC, and mDC between the groups (Fig 2B). The percentages of mDC-IL6 + significantly decreased in the high-VFA group compared with those in the low-VFA group (Fig 2C) and showed a positive correlation with pDC-IFNα+ (Fig 2D).

### Effects of VFA and pDC-IFNα+ on SARS-CoV-2 infection

**Fig 3:**
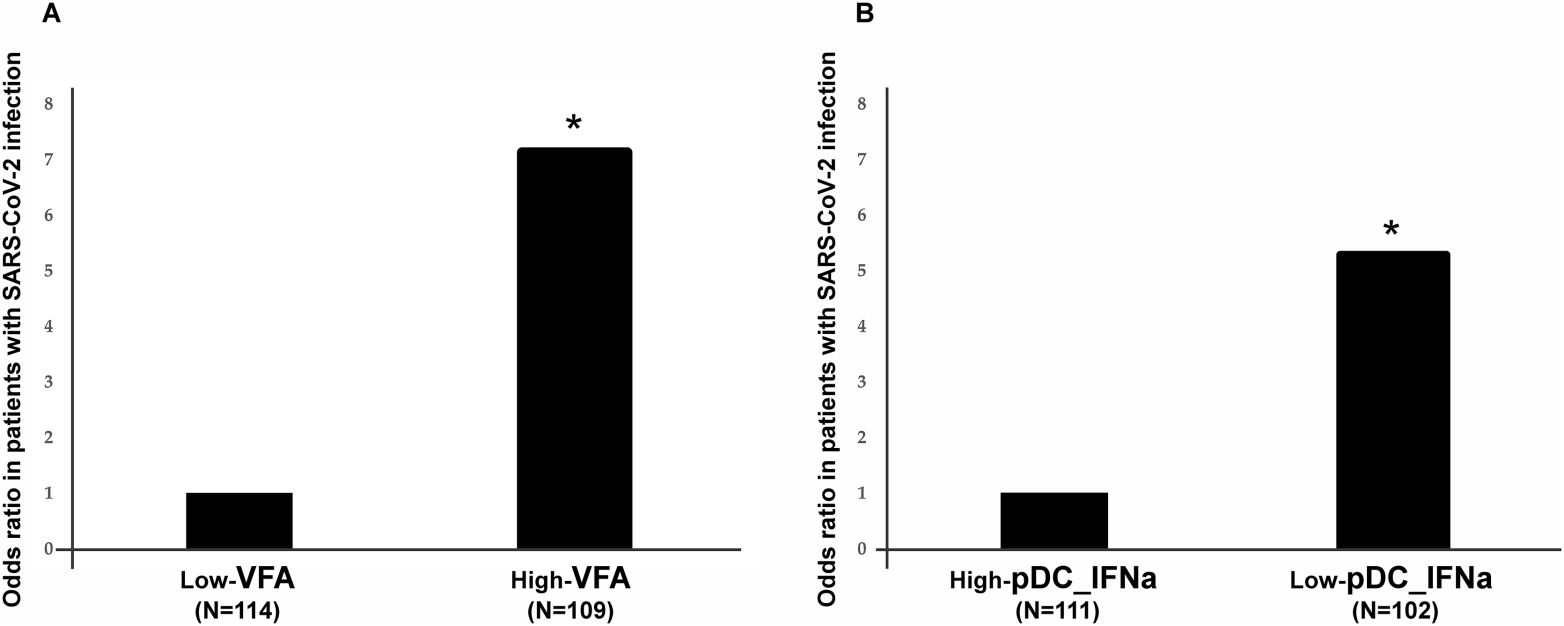
Odds ratio for VFA and pDC-IFNα in patients with SARS-CoV-2 infection. Odds ratio for VFA in patients with SARS-CoV-2 infection (A); odds ratio for pDC-IFNα in patients with SARS-CoV-2 infection (B). P-values were calculated using logistic regression by adjusting for age, sex, alcohol intake, exercise habits, smoking habits, the body mass index, and the number of vaccinations for SARS-CoV-2. ** P < 0.01.

VFA, visceral fat area; pDC-IFNα, plasmacytoid dendritic cell-derived interferon alpha; SARS-CoV-2, severe-acute-respiratory-syndrome-related coronavirus

The association between SARS-CoV-2 infection and VFA or pDC-IFNα was assessed (Fig 3A-3B). Various environmental factors, including age and sex, might affect the association between SARS-CoV-2 infection and VFA or pDC-IFNα + . These results may have been affected by the confounding variables. Therefore, we calculated the ORs and 95% CI for SARS-CoV-2 infection by assessing VFA or pDC-IFNα+ using logistic regression after adjusting for age, sex, muscle mass, education, smoking, the BMI, exercise habits, alcohol consumption, prevalent confounding diseases (hypertension, hyperlipidemia, and hyperglycemia), and the number of vaccinations for SARS-CoV-2 (Fig 3A-3B). After adjusting for confounding factors, VFA and pDC-IFNα + was found to be significantly associated with SARS-CoV-2 infection (OR, 7.1 and 95% CI, 2.8–17.8; P < 0.001 for VFA and OR, 5.2 and 95% CI, 2.7–10.3; P < 0.001 for pDC-IFNα+). These results suggest that individuals with high VFA or low pDC-IFNα were more susceptible to SARS-CoV-2, as expected.

### Synergistic effect of VFA and pDC-IFNα on SARS-CoV-2 infection

**Fig 4:**
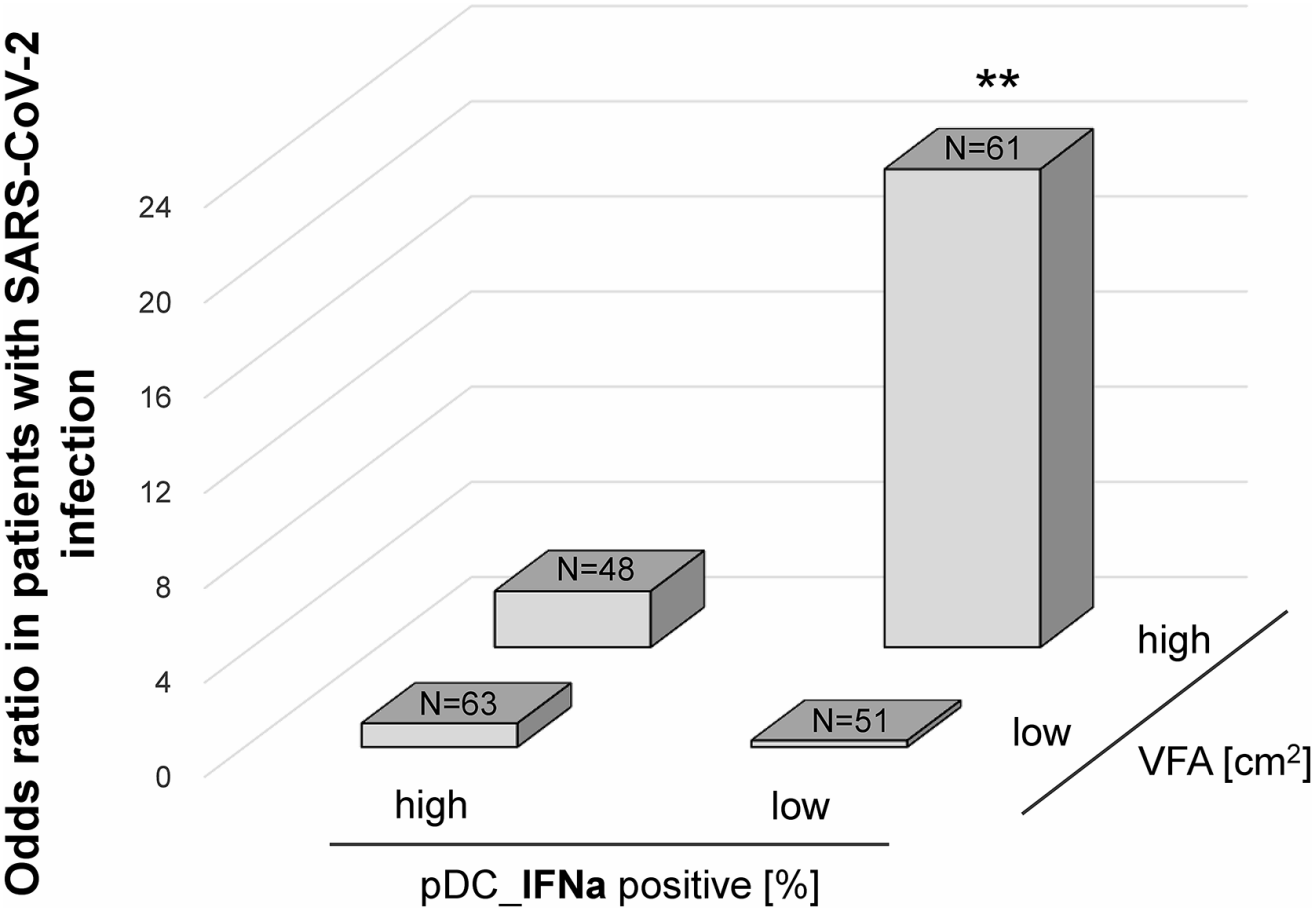
Odds ratio for the interaction between VFA and pDC-IFNα in patients with SARS-CoV-2. P-values were calculated using logistic regression adjusted for age, sex, alcohol intake, exercise habits, smoking habits, the BMI, and the number of vaccinations for SARS-CoV-2. ** P < 0.01. VFA, visceral fat area; pDC-IFNα, plasmacytoid dendritic cell-derived interferon alpha; SARS-CoV-2, severe-acute-respiratory-syndrome-related coronavirus; BMI, body mass index.

As shown in Fig 1A, although VFA and pDC-IFNα+ were significantly and negatively correlated (r = −0.27, P < 0.001), some individuals exhibited high VFA as well as high pDC-IFNα production, whereas others exhibited low VFA and pDC-IFNα production (Fig 1). Therefore, we next confirmed the synergistic effect of VFA and pDC-IFNα production on SARS-CoV-2 infection. Individuals were divided into four groups based on the median values of VFA and pDC-IFNα levels. The number of individuals in each group was as follows: low VFA_high pDC-IFNα group (n = 63), low VFA_low pDC-IFNα group (n = 51), high VFA_high pDC-IFNα group (n = 48), and high VFA_low pDC-IFNα group (n = 61). As shown in Fig 4, the ORs for each group were calculated using the low VFA_high pDC-IFNα group as a control, as this group was expected to be the least susceptible to SARS-CoV-2 infection (Fig 4A). Among the 223 participants, 16 had a history of SARS-CoV-2 infection and 5 had influenza virus infection, with no overlap between these two groups. Because the number of participants with influenza infection was small, an influenza-only analysis was not performed. Instead, SARS-CoV-2 and influenza infections were combined in Supplementary S2 Fig to assess whether the associations observed for SARS-CoV-2 were consistent when other viral infections were included. Only the high VFA_low pDC-IFNα group showed significant SARS-CoV-2 infection compared with the low VFA_high pDC-IFNα group (OR, 20.1 and 95% CI, 7.2–56.3; P = 0.003 for high VFA_low pDC-IFNα, OR, 2.4; P = n.s. for low pDC low VFA_low pDC-IFNα group and OR, 0.3; P = n.s. for high VFA_high pDC-IFNα group, Fig 4). Similar results were obtained in terms of the incidence of infectious diseases, including influenza and SARS-CoV-2 infection (OR, 19.0 and 95% CI, 7.0–51.7; P = 0.003 for high VFA_low pDC-IFNα group, OR, 1.3; P = n.s. for low pDC low VFA_low pDC-IFNα group and OR, 0.5; P = n.s. for high VFA_high pDC-IFNα group, S Fig 2).

## Discussion

To the best of our knowledge, this is the first study to investigate the association of VFA and IFN-I with SARS-CoV-2 infection. Previous studies have shown that obesity can result in reduced production of IFNα by PBMCs in response to TLR7 stimulation [24]; moreover, patients with obesity and SARS-CoV-2 infection have reduced serum levels of IFNα [25]. We observed a negative correlation between IFNα production from pDCs within PBMCs and the VFA. Moreover, increased levels visceral fat or decreased pDC-IFNα production were significantly associated with SARS-CoV-2 infection. Additionally, individuals with increased VFA and decreased pDC-IFNα production exhibited markedly higher OR for SARS-CoV-2 infection than the controls. These findings suggest that visceral fat and pDCs are important synergistic determinants of SARS-CoV-2 infection in individuals with visceral fat obesity. Although formal interaction analysis did not support a statistically significant sex difference in the association between VFA and pDC-derived IFNα production, the trend observed in females may reflect previously reported sex-related differences in pDC function, such as higher IFN-I production upon TLR7 stimulation in females compared with males [38,39]. This heightened baseline pDC activity in females could potentially make their immune response more sensitive to the metabolic disturbances associated with visceral fat accumulation. However, multivariable interaction analysis adjusting for relevant confounding factors did not identify a statistically significant sex-by-VFA interaction, indicating that we did not find sufficient evidence that the association between VFA and pDC-derived IFNα production differs by sex in this cohort.

Several previous studies have reported that pDC function is influenced by host factors such as obesity, sex, and aging. Obesity has been associated with impaired IFN-I responses in pDCs [24] while sex-related differences in pDC activity, including enhanced IFN-I production in females, have also been described [38,39]. Aging has also been reported to affect pDC function [40], although the present study focused on a relatively narrow age range of middle-aged individuals. Our findings are consistent with previous reports on obesity- and sex-related modulation of pDC function and extend these observations by demonstrating that visceral fat accumulation, which is not fully captured by BMI, is closely associated with reduced pDC-derived IFNα production and increased susceptibility to viral infection in a middle-aged population.

TLR7 and TLR8 are pivotal for detecting ssRNA from viruses, initiating innate immune responses. TLR7 is predominantly expressed in pDCs [13,41], whereas TLR8 is commonly found in mDCs [42]. Upon stimulation with specific TLR7/8 ligands, a notable decrease in IFNα production by pDCs and IL6 production by mDCs was observed in individuals with increased visceral adiposity, indicating a potential impairment in TLR7 and TLR8 signaling pathways. IFNα production primarily involves the transcription factor IRF7, whereas TNFα and IL6 production predominantly occurs via NF-κB activation following TLR7 engagement [43]. This suggests that IRF7-dependent signaling may be particularly susceptible to increased visceral adiposity. The mechanism underlying the selective reduction in IL6 production by mDCs remains unclear but may involve specific components of the IL6 signaling pathway.

Our finding of decreased IFN-α production in individuals with elevated visceral fat is consistent with that of prior studies that reported reduced IFN-α responses to TLR7 stimulation in PBMCs from individuals with obesity [24]. We identified pDCs within PBMCs as the main contributors to this reduction in IFN-α production in response to TLR7/8 stimulation associated with visceral fat obesity. Given that RNA viruses, including SARS-CoV-2 and influenza, are recognized by TLR7/8 [33,34], individuals with visceral fat obesity may exhibit compromised pDC-IFNα responses upon exposure to these viruses, potentially increasing their susceptibility to infections.

Although the mechanisms underlying reduced pDC-IFN-α production are not comprehensively understood, studies have observed that pDCs accumulate in the visceral fat of high-fat diet-induced obese mice, where they produce IFN-α even in the absence of infection [44]. This prolonged IFN-α production may lead to pDC exhaustion, reducing their responsiveness to viral antigens and weakening antiviral immunity. In individuals with high visceral fat, elevated serum CRP levels indicate a state of chronic, low-grade inflammation [45,46] without active infection, suggesting that similar mechanisms may occur in individuals with visceral adiposity. This chronic inflammatory environment may drive continuous low-level activation of pDCs, leading to prolonged IFN-α production, as observed in the murine model, and potentially contributing to pDC exhaustion in individuals with visceral adiposity.

The correlation between decreased mDC-derived IL-6 and reduced pDC-derived IFNα in individuals with increased visceral fat provides additional insights into the impact of obesity on immune function [47]. pDC-derived IFNα is essential for activating dendritic cell functions, including those of mDCs, which are critical for initiating adaptive immunity and promoting antibody responses against viral infections [48]. This suggests that reduced IFNα production in individuals with high visceral fat could impair mDC function, thereby potentially compromising adaptive immunity. Impaired mDC function may lead to inadequate antigen presentation, limiting T cell activation and ultimately reducing the body’s antiviral defenses. Although the precise mechanisms underlying the reduced IL-6 production by mDCs in individuals with increased VFA remain unclear, several components of the IL-6 production pathway may be involved. Notably, mDC-derived TNFα production was not altered, suggesting that visceral fat accumulation does not cause a global impairment of TLR7/8 signaling in mDCs. Instead, the selective reduction in IL-6 production may reflect differential regulation of cytokine outputs, as IL-6 production downstream of TLR7/8 stimulation requires not only NF-κB but also MAPK-dependent and metabolic pathways [49], and may therefore be more susceptible to alterations in cellular signaling and metabolic states associated with visceral fat accumulation. Future studies will be required to further clarify these mechanisms.

Both visceral fat and pDC-IFNα are critical in the control of viral invasion. However, no studies have examined the synergistic effect of VFA and pDC-IFNα against infectious diseases. To prevent unpredictable infectious diseases, controlling VFA and pDC-IFNα production is thought to be important; therefore, we examined the association of visceral fat and pDC-IFNα production with SARS-CoV-2 infection and found that individuals with high VFA or low pDC-IFNα production were more susceptible to SARS-CoV-2, as expected. As shown in Fig 2, there are individuals with high VFA but high pDC-IFNα production, or individuals with low VFA but low pDC-IFNα production. Therefore, we next confirmed the synergistic effect of VFA and pDC-IFNα production on SARS-CoV-2 infection and found that only the high VFA_low pDC-IFNα group exhibited SARS-CoV-2 infection when compared with the low VFA_high pDC-IFN group (OR, 19; P = 0.003, Fig 3A). This finding indicates that individuals with accumulated visceral fat and reduced pDC activity may be more susceptible to respiratory viral infections, such as SARS-CoV-2 and influenza virus. Individuals with visceral fat obesity are known to be in a chronic inflammatory state [46]. Furthermore, individuals with decreased pDC activity show increased SARS-CoV-2 severity [25,26] The individuals in the high VFA_low pDC-IFNα group had both conditions occurring at the same time; such individuals might be more susceptible to infection if they encounter the virus. Interestingly, previous trials have reported a significant improvement in the symptoms of SARS-CoV-2 infection following the administration of recombinant IFN-I [50]. These findings suggest that both maintaining lower visceral fat levels and enhancing pDC-derived IFNα responses may contribute to improved antiviral defense.

A limitation of the present study is that it was a cross-sectional study and not a longitudinal cohort study, and we could not assess whether the low VFA or high pDC-IFNα levels were a risk factor for future infections. As this study was performed in a limited country, region, and race, reproducibility should be confirmed in different countries and races. Although most participants (approximately 90%) had received SARS-CoV-2 vaccination before blood collection, detailed information on the timing of vaccination, the exact interval between SARS-CoV-2 or influenza infection and sample collection, and the severity of prior infections [51] was not available. In addition, information on infections occurring more than one year before the study was not collected. Therefore, potential confounding effects of vaccination timing, infection timing, or infection severity on IFNα responses or susceptibility to infection cannot be completely excluded, and the potential long-term effects of infection severity on pDC function could not be fully evaluated.

## Conclusions

We found that VFA was negatively correlated with the percentage of IFNα-producing pDCs. Furthermore, patients with high VFA or low pDC-IFNα production were found to be more susceptible to SARS-CoV-2 infection and influenza. These data suggest that maintaining a low VFA and preserving pDC-derived IFNα production may help reduce susceptibility to viral infections.

## Supporting information

S1 Fig Gating strategy for intracellular cytokine production by pDC and mDC in PBMC. After exclusion of dead cells, pDCs were defined as lineage⁻ HLA-DR ⁺ CD11c ⁻ CD123 ⁺ cells, and mDCs as lineage⁻ HLA-DR ⁺ CD11c ⁺ CD123 ⁻ cells. Non-pDCs were defined as CD123 ⁻ CD303 ⁻ cells. Gates were set using fluorescence minus one (FMO), isotype, and unstimulated full-stain controls.
(PDF)

S2 Fig Odds ratio for the interaction between VFA and pDC-IFNα in patients with SARS-CoV-2 or influenza infection. Odds ratio of interaction of VFA and pDC-IFNα in patients with SARS-CoV-2 and influenza infections. P-value was calculated using logistic regression by adjusting for age, sex, alcohol intake, exercise habits, smoking habits, the BMI, number of vaccinations for SARS-CoV-2, and number of vaccinations for influenza. ** P < 0.01. VFA, visceral fat area; pDC-IFNα, plasmacytoid dendritic cell-derived interferon alpha; SARS-CoV-2, severe-acute-respiratory-syndrome-related coronavirus; BMI, body mass index.
(PDF)

S3 Fig Sex-stratified Spearman correlation analysis of VFA and pDC-derived IFNα production. Scatter plots showing the relationship between VFA and pDC-derived IFNα production in (A) women and (B) men. Linear regression lines are shown for visualization purposes. Correlations between the two variables were determined using Spearman’s correlation coefficient. VFA, visceral fat area; pDC-IFNα, plasmacytoid dendritic cell-derived interferon alpha.
(PDF)

S1 Table Characteristics of the participants divided into two groups based on the median values of VFA. * P < 0.05; ** P < 0.01; *** P < 0.001. Data represent mean (standard deviation). P-values were evaluated between the low-VFA and high-VFA groups. a Wilcoxon rank-sum test was performed. b Fisher’s exact test was performed. VFA, visceral fat area; SBP, systolic blood pressure; DBP, WC, waist circumference; HbA1C, hemoglobin A1c; CRP, C-reactive protein.
(DOCX)

S2 Table Characteristics of the participants divided into two groups based on the median values of pDC-IFNa * P < 0.05; ** P < 0.01; *** P < 0.001. Data represent mean (standard deviation). P-values were evaluated between the low-pDC and high-pDC-IFNα groups. a Wilcoxon rank-sum test was performed. b Fisher’s exact test was performed. pDC-IFNα, plasmacytoid dendritic cell-derived interferon alpha; SBP, systolic blood pressure; DBP, WC, waist circumference; HbA1C, hemoglobin A1c; CRP, C-reactive protein.
(DOCX)
