# Supplementary figures and images for: Association of visceral fat and plasmacytoid dendritic cell-derived interferon alpha with SARS-CoV-2 infection
Source: PLoS One. 2026 Apr 10;21(4):e0344870. doi: 10.1371/journal.pone.0344870 (PMC13068220; doi:10.1371/journal.pone.0344870)

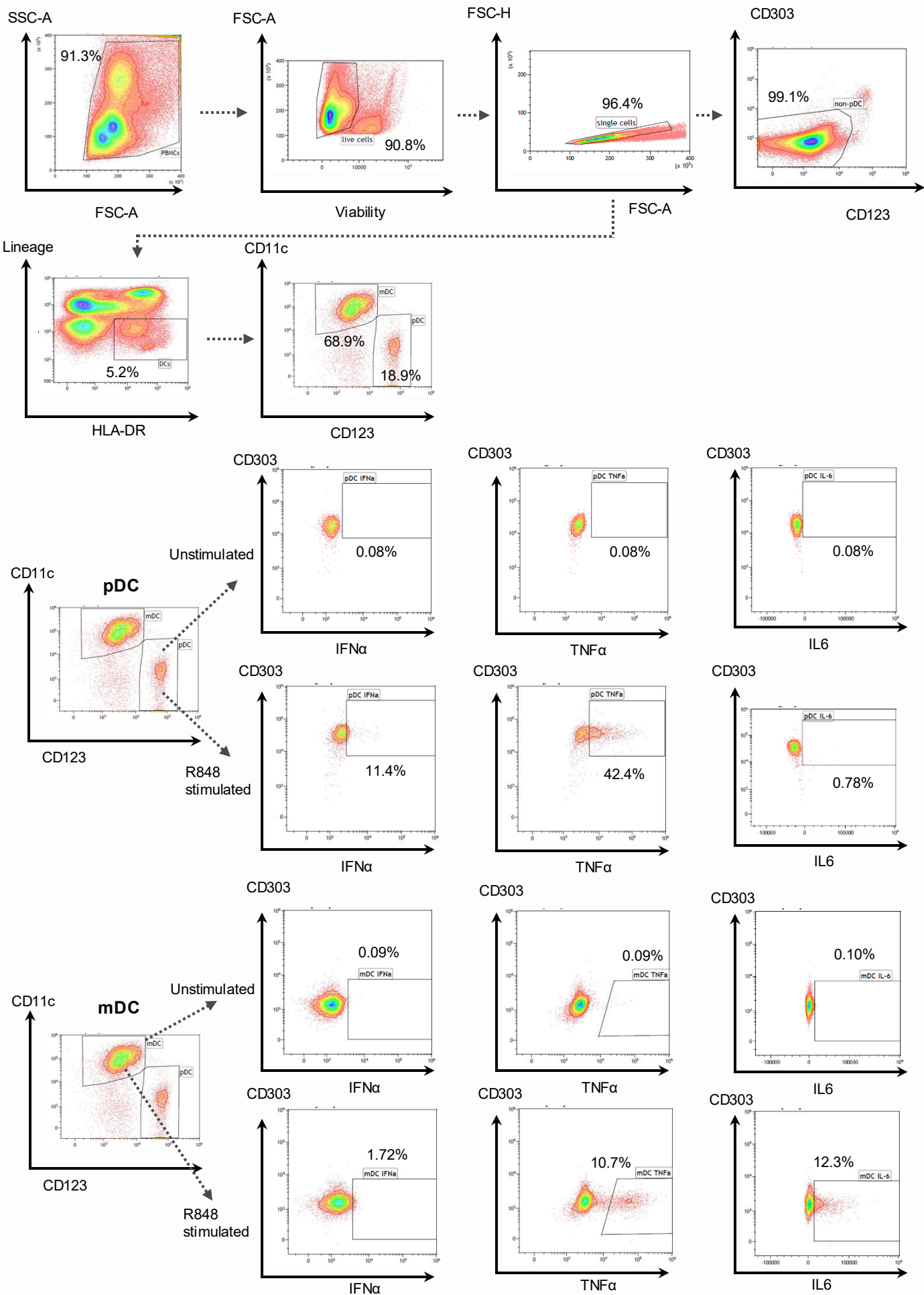

**S1 Fig. Gating strategy for intracellular cytokine production by pDC and mDC in PBMC.**

Supplement: S1 Fig — After exclusion of dead cells, pDCs were defined as lineage⁻ HLA-DR ⁺ CD11c ⁻ CD123 ⁺ cells, and mDCs as lineage⁻ HLA-DR ⁺ CD11c ⁺ CD123 ⁻ cells. Non-pDCs were defined as CD123 ⁻ CD303 ⁻ cells. Gates were set using fluorescence minus one (FMO), isotype, and unstimulated full-stain controls. (PDF) [file pone.0344870.s001.pdf]

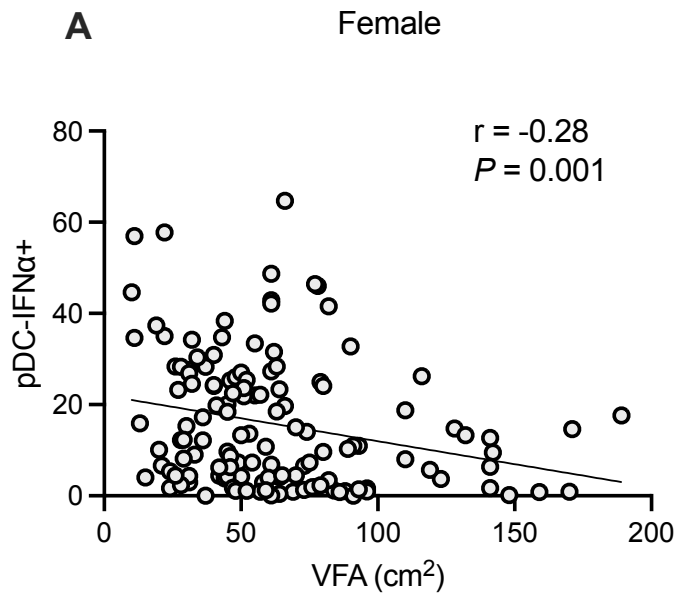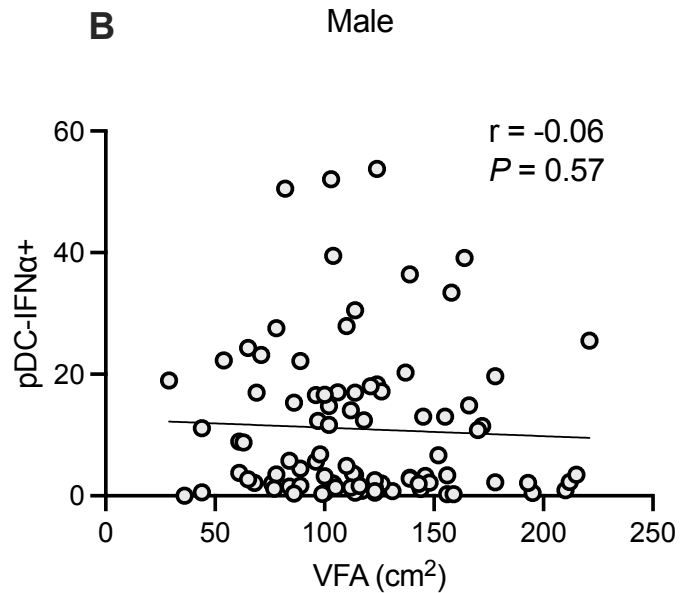

**S3 Fig. Sex-stratified Spearman correlation analysis of VFA and pDC-derived IFNα production.**

Supplement: S3 Fig — Scatter plots showing the relationship between VFA and pDC-derived IFNα production in (A) women and (B) men. Linear regression lines are shown for visualization purposes. Correlations between the two variables were determined using Spearman’s correlation coefficient. VFA, visceral fat area; pDC-IFNα, plasmacytoid dendritic cell-derived interferon alpha. (PDF) [file pone.0344870.s003.pdf]
