# Supplementary material for: Association of visceral fat and plasmacytoid dendritic cell-derived interferon alpha with SARS-CoV-2 infection
Source: PLoS One. 2026 Apr 10;21(4):e0344870. doi: 10.1371/journal.pone.0344870 (PMC13068220; doi:10.1371/journal.pone.0344870)
Supplement: S2 Fig — Odds ratio of interaction of VFA and pDC-IFNα in patients with SARS-CoV-2 and influenza infections. P-value was calculated using logistic regression by adjusting for age, sex, alcohol intake, exercise habits, smoking habits, the BMI, number of vaccinations for SARS-CoV-2, and number of vaccinations for influenza. ** P < 0.01. VFA, visceral fat area; pDC-IFNα, plasmacytoid dendritic cell-derived interferon alpha; SARS-CoV-2, severe-acute-respiratory-syndrome-related coronavirus; BMI, body mass index. (PDF) [file pone.0344870.s002.pdf]

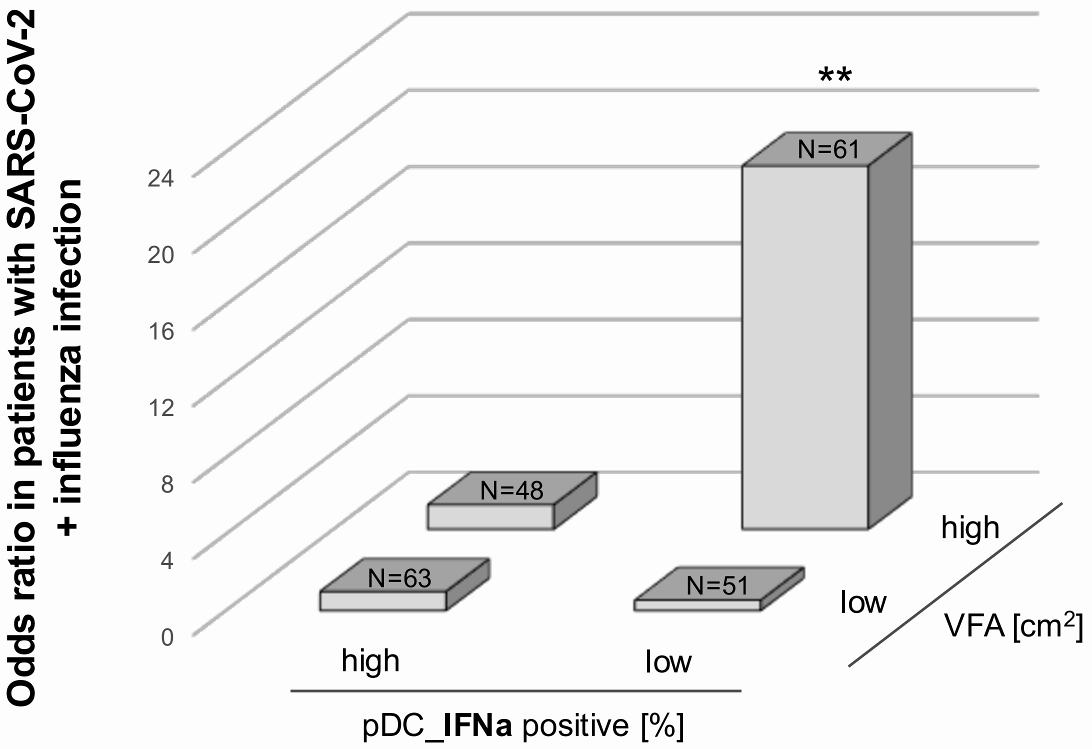

S2 Fig. Gating strategy for intracellular cytokine production by pDC and mDC in PBMC.
