# Supplementary material for: Association of visceral fat and plasmacytoid dendritic cell-derived interferon alpha with SARS-CoV-2 infection
Source: PLoS One. 2026 Apr 10;21(4):e0344870. doi: 10.1371/journal.pone.0344870 (PMC13068220; doi:10.1371/journal.pone.0344870)
Supplement: S1 Table — * P < 0.05; ** P < 0.01; *** P < 0.001. Data represent mean (standard deviation). P-values were evaluated between the low-VFA and high-VFA groups. a Wilcoxon rank-sum test was performed. b Fisher’s exact test was performed. VFA, visceral fat area; SBP, systolic blood pressure; DBP, WC, waist circumference; HbA1C, hemoglobin A1c; CRP, C-reactive protein. (DOCX) [file pone.0344870.s004.docx]

**S1 Table.** Characteristics of the participants divided into two groups based on the median values of VFA

|  | Low-VFA | High-VFA | P value ^a, b^ |
| --- | --- | --- | --- |
| n | 114 | 109 |  |
| DBP (mean (SD)) | 76.5 (5.6) | 91.9 (7.7) | <0.001^***^ |
| SBP (mean (SD)) | 121.0 (17.0) | 132.9 (16.4) | <0.001^***^ |
| WC (mean (SD)) | 75.9 (10.0) | 85.1 (10.3) | <0.001^***^ |
| Glucose (mean (SD)) | 90.4 (15.7) | 100.8 (18.7) | <0.001^***^ |
| Insulin (mean (SD)) | 4.8 (2.1) | 8.0 (4.8) | <0.001^***^ |
| HbA1C (mean (SD)) | 5.4 (0.5) | 5.6 (0.6) | <0.001^***^ |
| CRP (mean (SD)) | 0.031 (0.036) | 0.123 (0.211) | <0.001^***^ |
| Smoking habit (Yes) (n, %) | 15 (13%) | 27 (25%) | 0.041^*^ |
| Exercise habit (Yes) (n, %) | 21 (18%) | 23 (21%) | 0.738 |
| Alcohol drinking (g/day) | 10.0 (19.0) | 17.5 (23.0) | 0.005^**^ |
| Hypertension (Yes) (n, %) | 29 (25%) | 64 (59%) | <0.001^***^ |
| Hyperglycemia (Yes) (n, %) | 3 (2.6%) | 15 (14%) | 0.003^**^ |
| Dyslipidemia (Yes) (n, %) | 18 (16%) | 44 (40%) | <0.001^***^ |
| Education (college or higher) (Yes) (n, %) | 10 (8.8%) | 15 (14%) | 0.290 |

* P < 0.05; ** P < 0.01; *** P < 0.001. Data represent mean (standard deviation). P-values were evaluated between the low-VFA and high-VFA groups. ^a^ Wilcoxon rank-sum test was performed. ^b^ Fisher’s exact test was performed. VFA, visceral fat area; SBP, systolic blood pressure; DBP, WC, waist circumference; HbA1C, hemoglobin A1c; CRP, C-reactive protein
