# Supplementary material for: Association of visceral fat and plasmacytoid dendritic cell-derived interferon alpha with SARS-CoV-2 infection
Source: PLoS One. 2026 Apr 10;21(4):e0344870. doi: 10.1371/journal.pone.0344870 (PMC13068220; doi:10.1371/journal.pone.0344870)
Supplement: S2 Table — Data represent mean (standard deviation). P-values were evaluated between the low-pDC and high-pDC-IFNα groups. a Wilcoxon rank-sum test was performed. b Fisher’s exact test was performed. pDC-IFNα, plasmacytoid dendritic cell-derived interferon alpha; SBP, systolic blood pressure; DBP, WC, waist circumference; HbA1C, hemoglobin A1c; CRP, C-reactive protein. (DOCX) [file pone.0344870.s005.docx]

**S2 Table.** Characteristics of the participants divided into two groups based on the median values of pDC-IFNa

|  | Low-pDC_IFNa | High-pDC_IFNa | P value ^a, b^ |
| --- | --- | --- | --- |
| n | 112 | 111 |  |
| DBP (mean (SD)) | 85.8 (9.7) | 82.3 (10.5) | 0.008^*^ |
| SBP (mean (SD)) | 127.0 (16.2) | 126.6 (19.1) | 0.583 |
| WC (mean (SD)) | 80.9 (10.2) | 79.9 (12.0) | 0.254 |
| Glucose (mean (SD)) | 96.8 (17.7) | 94.3 (18.2) | 0.061 |
| Insulin (mean (SD)) | 6.3 (3.6) | 6.5 (4.4) | 0.938 |
| HbA1C (mean (SD)) | 5.5 (0.5) | 5.4 (0.5) | 0.017^*^ |
| CRP (mean (SD)) | 0.078 (0.139) | 0.074 (0.173) | 0.120 |
| Smoking habit (Yes) (n, %) | 26 (23%) | 16 (14%) | 0.131 |
| Exercise habit (Yes) (n, %) | 20 (18%) | 24 (22%) | 0.591 |
| Alcohol drinking (g/day) | 13.4 (20.3) | 14.0 (22.4) | 0.496 |
| Hypertension (Yes) (n, %) | 50 (45%) | 43 (39%) | 0.416 |
| Hyperglycemia (Yes) (n, %) | 11 (9.8%) | 7 (6.3%) | 0.462 |
| Dyslipidemia (Yes) (n, %) | 36 (32%) | 26 (23%) | 0.179 |
| Education (college or higher) (Yes) (n, %) | 14 (13%) | 11 (9.9%) | 0.672 |

* P < 0.05; ** P < 0.01; *** P < 0.001. Data represent mean (standard deviation). P-values were evaluated between the low-pDC and high-pDC-IFNα groups.

^a^ Wilcoxon rank-sum test was performed. ^b^ Fisher’s exact test was performed.

pDC-IFNα, plasmacytoid dendritic cell-derived interferon alpha; SBP, systolic blood pressure; DBP, WC, waist circumference; HbA1C, hemoglobin A1c; CRP, C-reactive protein
